# Chemical composition and antioxidant content of *Thymus vulgaris* honey and *Origanum vulgare* essential oil; their effect on carbon tetrachloride-induced toxicity

**DOI:** 10.14202/vetworld.2021.292-301

**Published:** 2021-01-30

**Authors:** Hamada Imtara, Noori Al-Waili, Abderrazak Aboulghazi, Abdelfattah Abdellaoui, Thia Al-Waili, Badiaa Lyoussi

**Affiliations:** 1 Department of Biology and Biotechnology, Faculty of Arts and Sciences, Arab American University Palestine, P.O. Box 240, Jenin, State of Palestine; 2 New York Medical Care for Nephrology, New York, USA; 3 Laboratory of Physiology, Pharmacology and Environmental Health, Faculty of Sciences Dhar El Mehraz, BP 1796 Atlas, University Sidi Mohamed Ben Abdallah, Fez 30 000, Morocco.

**Keywords:** antioxidants, carbon tetrachloride, *Origanum vulgare* essential oil, thymus honey, toxicity

## Abstract

**Aim:**

The study investigated the chemical composition, antioxidant content, and antioxidant activity of *Thymus vulgaris* honey (TVH) and *Origanum vulgare* essential oil (OVEO) and their mixture effect on carbon tetrachloride (CCl_4_)-induced toxicity.

**Materials and Methods:**

The study conducted physicochemical characterization and chemical analysis of TVH and OVEO with the use of gas chromatography–mass spectrometry and high-performance liquid chromatograph (HPLC). The antioxidant activity of TVH and OVEO was done with the use of 1,2-diphenyl-1-picrylhydrazyl (DPPH) radical scavenging activity. The study used nine groups of rats to investigate the effect of TVH, OVEO, and a mixture of TVH and OVEO (HEM) on CCl_4_-induced toxicity. Intraperitoneal injection of CCl_4_ (1 mL/100 g) was used to induce toxicity. The doses of TVH and OVEO were 1 mg/kg.b.wt, and 50 mg/kg.b.wt, respectively. HEM contains TVH (1 mg/kg.b.wt) and OVEO (50 mg/kg.b.wt).

**Results:**

TVH has a high content of phenols, flavonoids, and flavanols. HPLC analysis showed that TVH contains, for the 1^st^ time, epicatechin gallate, and at a high concentration. OVEO includes a high percentage of carvacrol and thymol. With the use of DPPH, OVEO was more potent than TVH. CCl_4_ caused significant liver and kidney damage and lipid disorders, which were alleviated by HVT, OVEO, and HEM. HVT was more potent than OVEO (p<0.05), and HEM was more potent than HVT and OVEO (p<0.05).

**Conclusion:**

The study identified high content of epicatechin gallate for the 1^st^ time in TVH, and OVEO contains a high percentage of thymol and carvacrol. Epicatechin gallate might be useful as a marker for TVH. Mixing OVEO and TVH significantly potentiated their protection against CCl_4_-induced liver and kidney toxicity.

## Introduction

A large amount of data showed that honey has biological activities such as antimicrobial, anti-inflammatory, antioxidant, and immune-modulatory activities [1-3]. *Thymus vulgaris* honey (TVH) and *Origanum vulgar* essential oil (OVEO) have important biological activities and have great potential to be introduced in the illness treatment. TVH is unifloral honey, which has antimicrobial activity and wound healing properties. It is useful in managing radiation-induced mucositis and xerostomia in head and neck cancer patients [4-6]. Honey has been mentioned in Holy books, the Talmud, the Bible, and the Quran as a healer of human diseases. In the Surat Al-Nahel (the Bee chapter), it says (translating the meaning): (*And your LORD taught the bee to build its cells in the mountains, on the trees, and in men’s habitations, then to eat of all the fruits of the earth and find with skill the spacious paths of its LORD, their issues from within their bellies, a drink of varying colors, wherein is healing for men, verily in this is a sign for those who give thought*)*. O. vulgar* is one of the species of *Origanum*. Herbs of this family are used to improve the flavor of foods. A recent review showed that *O vulgar* has an anticancer, anti-inflammatory, antiproliferative, antibacterial, and hepatoprotective effect [7]. The kidney and liver are vital organs with a wide range of biological functions that include metabolism, transforming, transport, and clearance of toxic substances. Furthermore, the kidney affects blood electrolytes, bone metabolism, and bone marrow function, and the liver is involved in lipid metabolism. Carbon tetrachloride (CCl_4_) is a volatile organic alkyl halogen, which is metabolized in the liver and generates trichloromethyl (CCl_3_) and trichloromethyl peroxyl (CCl_3_O_2_).

CCl_4_ is very toxic to the liver and kidney and used experimentally to induce liver and kidney damage. Others and we have found that honey has a protective effect against CCl_4_-induced liver and kidney toxicity [8-10]. Furthermore, honey increases urine output and creatinine clearance, and it has protection against lead-induced kidney and liver toxicity [11,12]. The hepatoprotective activity of *O. vulgare* leaves extract against CCl_4_-induced hepatotoxicity in rats has been reported [13]. However, no study investigated the effect of TVH or OVEO on CCl_4_-induced liver and kidney toxicity. Therefore, the present study examined the protective effect of TVH, OVOE, and their combination (HEM) against CCl_4_-induced toxicity in rats. It was well known that the chemical composition of various honey samples is different [14,15]. Therefore, the authors designed the study to identify the chemical components and antioxidant activity of OVEO and TVH collected in Morocco.

The study showed for the first time the presence of epicatechin gallate in TVH collected in Fez, Boulemane, Morocco, and the ability of TVH, OVEO, and HEM to protect against CCl_4_-induced liver and kidney damage.

## Materials and Methods

### Ethical approval

The ethical institutional committee, Faculty of Sciences Dhar El Mahraz, University Sidi Mohamed Ben Abdallah, Fez, Morocco, approved the protocol. All the experimental proceedings achieved in laboratory animals followed the internationally accepted standard guidelines for animal care. The authors tried to minimize animal suffering and the number of animals used.

### Study period and location

This study was conducted in June month of 2017 at Sidi Mohamed Ben Abdellah University, Morocco.

### Honey samples

The authors purchased honey (*T. vulgaris*, Zaitra; Arabic name) from beekeepers, Fez, Boulemane, Morocco. The honey sample was collected in 2015 and stored at room temperature (22-24°C) until analysis.

### Essential oil extraction

The aerial part of *O. vulgare* L plant was bought from the herbalist, Imouzzer, Boulemane, Morocco. The extraction of the essential oil was carried out by hydro-distillation in a Clevenger-type apparatus and was stored at 4°C in the presence of anhydrous sodium sulfate.

### The physicochemical and antioxidant content of honey

Free acidity, pH, ash, electrical conductivity, and moisture were measured in the TVH samples. The measurements were carried out according to the International Honey Commission IHC [16].

The color and melanoidins content was determined according to the method described by Naab *et al*. [17]. The mineral content was measured according to the method described by Terrab *et al*. [18]. The total phenol content was determined according to the method described by Singleton [19]. The result of phenol content was expressed as the mg gallic acid/100 g of honey. The total flavonoid content was determined according to the method described by Samatha *et al*. [20]. The flavonoid content was expressed as mg quercetin/100 g of honey (mg Eq Q/100 g). Total flavonol content was determined according to the method described by Sugathakumar *et al*. [21]. The flavonol content was expressed as mg quercetin equivalent per 100 g of the honey mass (mg Eq Q/100 g). The total antioxidant capacity was estimated by the phosphomolybdenum method according to the procedure described by Prieto *et al*. [22]. Total antioxidant capacity content was expressed as mg of ascorbic acid equivalent per 100 g of the honey mass (mg AA/100g).

### Antioxidant activity of TVH and OVEO

The antioxidant activity of TVH and OVEO was determined with the use of 1,2-diphenyl-1-picrylhydrazyl (DPPH) radical scavenging activity according to the method by Brand-Williams *et al*. [23]. Briefly, the ethanol solution of DPPH solution was added to dilution series of TVH and OVEO ranging from 15 to 500 for TVH and 0.8 to 100 mg/mL for OVEO and then incubated in the dark for 1 h. The absorbance was measured at 517 nm. The experiment was performed in triplicates, and average absorption was recorded for each concentration. Butylated hydroxytoluene (BHT) was used as a standard. The percent inhibition of the DPPH radical by the samples was calculated based on the following formula:

(The absorbance of negative control - The absorbance of the sample)/the absorbance of negative control)×100.

Based on each sample, IC_50_ was determined (concentration of samples able to scavenge 50% of DPPH free radicals).

### Identification of phenolic compounds of TVH by high-performance liquid chromatograph (HPLC)

The honey sample was subjected to base hydrolysis and extracted with ethyl acetate (liquid-liquid extraction) described by Aljadi and Yousoff [24]. In brief, 10 g of honey were dissolved in acidified distal water, and the phenolic compounds were extracted with the use of ethyl acetate. The phenolic extract was passed in the rotavapor to remove the solvent, and then the dry phenolic extract was dissolved in 5 mL of methanol grade HPLC. With the use of HPLC, the analyses were carried out at 280 nm for phenolic compounds and operated at 30°C using a C18 column (4.6 mm×150 mm)×5 mm in a thermal fisher apparatus. The injected volume was 20 mL. Pure compounds that were used as standards included caffeic acid, gallic acid, epicatechin gallate, coumaric acid, rosmarinic acid, ferulic acid, syringic acid, tannic acid, and pyrogallol. All the standards were dissolved in methanol and injected under the same chromatographic conditions as the honey extracts. Phenolic compounds of honey were identified by comparing their retention times with those of pure standards. The results were obtained in mg/100 g of honey.

### Gas chromatography–mass spectrometry (GC/MS) analysis of OVEO

Regarding GC/MS analysis, the OVOE sample was diluted in hexane with a dilution of 10:100. The analysis of volatile constituents was completed using GC/MS method (GC ULTRA S/N 20062969; Polaris QS/N 210729), equipped with an HP-5MS column nonpolar fused silica (60 m×0.32 mm, 0.25 mm thickness). The temperature of the injector was 250°C. The column temperature was programmed from 40 to 260°C at 2°C/min. The carrier gas was helium, the flow rate was 1 mL/min, and the volume of sample injected was 1 μL of diluted oil. The identification of the components has been made by determination of their indices of retention Kovats index (Ki) compared to a Ki of a series of n-alkanes (C8-C20) and by comparing their mass spectra recorded with those stored in the database of the spectrometer (NIST MS Library v. 2.0, US Department of Commerce, California, USA), and the literature [25].

### Effect of TVH, OVEO, and their mixture in CCl_4_-induced toxicity

#### Animals

Forty-five adult male Wister rats (221±22 g) were used. The animals were kept in a diffusely lit and temperature-controlled room with a diurnal 12 h light cycle, where the temperature (25±1°C) and relative humidity (55±5%) were kept constant. The animals were given free access to a standard laboratory diet and water before the experiments.

#### Preparation of honey-essential oil mixture

OVEO was dissolved in tween 80, and TVH was dissolved in water, and they mixed with each other. One mL of HEM containing TVH (1 g/kg.b.wt) and OVEO (50 mg/kg.b.wt) was used and delivered to the animals by gavage.

#### Study design

The experimental animals received intraperitoneal injections of CCl_4_ (1 mL/100 g.b.wt) dissolved in olive oil, 10% solution, twice per week for 2 weeks. The administration of TVH, OVEO, and HEM was started on the 1^st^ day of CCl_4_ administration and was continued for 2 weeks.

The animals were randomly divided into nine groups, five animals each, as follows:

- Group I (water): The animals received drinking water 1 mL/kg.b.wt every day by gavage
- Group II (Tween 80): The animals received Tween 80 (2%) by gavage (1 mL/kg.b.wt) every day
- Group III (TVH): The animals received TVH by gavage (1 g/kg.b.wt) every day
- Group IV (OVEO): The animals received OVEO by gavage (50 mg/ kg.b.wt) every day
- Group V (HEM): The animals received HEM 1 mL/kg.b.wt by gavage every day
- Group VI (CCl_4_): The animals received CCl_4_
- Group VII (CCl_4_+ TVH): The animals received CCl_4_ and treated with TVH by gavage at a dose of 1 g/kg.b.wt every day
- Group VIII (CCl_4_ + OVEO): The animals received CCl_4_ and treated with OVEO by gavage at a dose of 50 mg /kg.b.wt every day
- Group IX (CCl4 + HEM): The animals received CCl4 and treated with HEM by gavage every day.

The doses of TVH, OVEO, and Tween 80 were used according to the previous publications [12,26,27]. At the end of the treatment period (2 weeks), the animals were sacrificed. Blood samples were collected in centrifuge tubes without anticoagulants and allowed to clot. The clotted blood was centrifuged at 4000× g for 20 min. Serum was separated for biochemical analyses.

### Statistical analysis

Results were expressed as mean ± SD. Statistical analysis was carried out by ANOVA with the use of GraphPad Prism (San Diego, California, USA) 6.0 program. p*<*0.05 was considered significant.

## Results

### Physicochemical properties of HTV and OVEO

The physicochemical parameters of honey showed that the pH was 3.90±0.01; free acidity was 37.93±2.25 mEq/kg, moisture was 16.4±0.01%, electric conductivity was 449±1731 mS/cm, ash was 0.24±0.01 %, and melanoidin was 1.10±0.02. The Pfund scale value was 0.33±0.01, which indicated that the color was dark amber.

Mineral content analysis of honey showed that the concentration of potassium was 829.55±0.51 mg/kg, sodium was 256.32 ± 0.15 mg/kg, magnesium was 61.47±0.21 mg/kg, and calcium was 166.22±0.09 mg/kg. OVEO analysis showed that the pH was 5.02, density was 0.9484 g/mL, and the refractive index was 1.5153.

### Phenol, flavonoid, and flavonol content of TVH

The phenol content value was 74.05±1.21 mg AGE/100 g, flavonoids content was 59.34±1.08 mg Eq Q/100g, and flavonol was 15.08±0.04 mg Eq Q/100 g.

**Figure-1:**
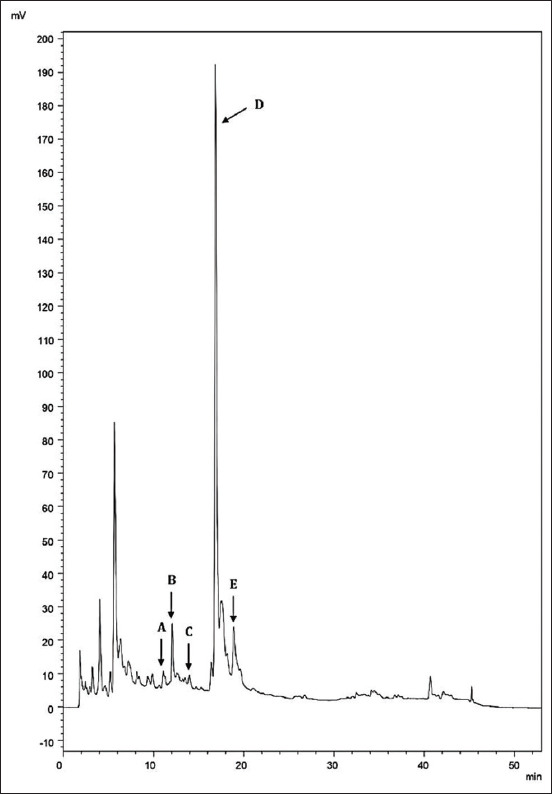
Chromatogram of *Thymus* vulgaris honey with identified standard compounds. A: Caffeic acid; B: Ferulic acid; C: Gallic acid; D: Epicatechin gallate; E: Pyrogallol.

The honey was analyzed through HPLC under the same chromatographic conditions (Figure-1). The analysis showed that the concentration of caffeic acid was 0.033±0.00 mg/100 g, ferulic acid was 0.68±0.021mg/100 g, gallic acid was 2.86±0.046 mg/100 g, epicatechin gallate was 6.91±0.05 mg/100 g, and pyrogallol was 3.5±0.09 mg/100 g. The analysis of honey did not show syringic acid, tannic acid, coumaric acid, or rosmoric acid.

### Antioxidant activity of TVH and OVEO

The total antioxidant capacity of TVH was 163.51±8.55 mg Eq AA/g. The IC50 value of BHT was 0.009±0.0001 mg/mL in the DPPH assay. However, the IC_50_ amount of OVEO was 0.30±0.02 mg/mL and of TVH was 10.85±0.02, which means that the DPPH scavenging activity of OVEO was higher than that of TVH (p<0.05).

### GC/MS analysis OVEO

Table-1 showed the results of the GC/MS analysis of OVEO. The study showed the presence of 26 compounds, and 92.45 % of essential oil has been identified. The major constituents of the oil were carvacrol (48.38%), thymol (26.55%), γ-terpinene (7.9%), and 1,8-Cineol (4.86%).

**Table-1:** Chemical composition of *Origanum vulgare* essential oil from Morocco.

Compounds	% Area	Kovats index
α-Pinene	0.10	948
2-Carene	0.15	1002
α-Phellandrene	0.03	1007
3-Carene	0.30	1009
1,4-Cineol	0.06	1012
α-Terpinene	0.81	1018
1,8-Cineol	4.86	1038
Cis-Ocimene	0.03	1072
γ-Terpinene	7.90	1084
Terpinolene	0.06	1096
Isoborneol	0.59	1158
β-terpineol	0.07	1162
Borneol	0.23	1172
4-terpinol	0.15	1235
Cis-carveol	0.07	1289
Thymol methyl ether	0.29	1325
Pulegone	0.05	1335
Thymol	26.55	1375
Carvacrol	48.38	1394
Caryophyllene	1.03	1426
α-Himachalene	0.15	1475
Longifolene-(V4)	0.06	1495
α-Cadinene	0.05	1538
γ-Muurolene	0.11	1542
γ-Cadinene	0.28	1571
Caryophyllene oxide	0.09	1628
Total	92.45	

### The effect of TVH, OVEO, and HEM on CCl_4_-induced toxicity

The effect of TVH, OVOE, and HEM on kidney and liver function and lipid profile in CCl_4_-untreated rats is demonstrated in Table-2. TVH decreased aspartate aminotransferase (AST) and alanine aminotransferase (ALT) (p<0.05) significantly. TVH and OVEO did not cause significant changes in the total protein, total bilirubin, direct bilirubin, lipid parameters, kidney function, and potassium and calcium level. However, OVEO caused a significant elevation of sodium level compared to the control group (p<0.05). HEM caused a significant lowering of AST, ALT, alkaline phosphatase (AP), and significantly increased total protein compared to the control group (p<0.05). Regarding the renal function and electrolytes, TVH, OVEO, and HEM did not cause significant changes in blood urea, creatinine, sodium, potassium, and calcium. Lipid assay showed that HEM lowered total cholesterol (TC), low-density lipoproteins (LDL), and very LDL (VLDL) and elevated high-density lipoprotein (HDL) compared to the control; the changes in TC level were significant (p<0.05).

**Table-2:** Effect of *Thymus vulgaris* honey, *Origanum vulgar* essential oil and HEM on liver, kidney function, and lipid profile in CCl4-untreated rats.

Variables	Treatment groups					F/P value

	Control	Tween 80	*Thymus vulgaris* honey	*Origanum vulgar* essential oil	HEM	
AST (U/L)	131.0±2.6	140.0±7.7*	120.0±3.4*#	123.0±3#	100.0±3.0*#+β	57.81/0.000
ALT (U/L)	124.0±2.9	133.0±4.7	112.0±9.0*#	126.0±1.5#+	109.0±4.63*#β	18.66/0.00
ALKP (U/L)	137.0±4.5	147.0±3.4*	139.0±3.4#	141.0±2.8	122.0±2.0*#+β	39.03/0.00
Total protein (g/dl)	6.5±0.07	6.4.0±0.22	6.6±0.15	6.3±0.15+	6.8±0.11*#β	8.37/0.0004
Total Bilirubin (mg/dl)	0.15±0.07	0.20±0.03	0.14±0.01	0.17±0.05	0.11±0.02#	3.21/0.034
Direct Bilirubin (mg/l)	0.1±0.02	0.13±0.06	0.09±0.04	0.11±0.02	0.08±0.03	1.34/0.28
Cholesterol (mg/dl)	71.0±10.0	80.0±6.0	71.0±8.0	84.0±2.0	52.0±7.0*#+β	15.04/0.00
Triglycerides (mg/dl)	39.0±6.0	43.0±5.0	37.0±11.0	47.0±25.0	26.0±9.0β	3.217/0.034
HDL (mg/dl)	22.0±2.0	20.0±2.0	22.0±3.0	22.0±4.0	24.0±1.0	1.47/ 0.248
LDL (mg/dl)	41.0±9.0	52.0±12.0	42.0±8.0	53.0±16.0	23.0±8.0#β	5.98/0.002
VLDL (mg/dl)	8.0±1.0	9.0±1.0	7.0±2.0	9.0±5.0	5.0±2.0	2.00/0.133
Creatinine (mg/dl)	0.34±0.04	0.39±0.01	0.38±0.04	0.38±0.03	0.34±0.02	2.30/0.93
Urea (mg/dl)	24.0±2.0	26.0±2.0	23.0±1.0	24.0±3.0	20.0±1.0*#β	6.315/0.019
Sodium (mmol/l)	127.0±2.6	128.0±3.6	129.0±1.5	136.0±4.9*#	129.0±5.8	3.987/0.014
Potassium (mmol/l)	4.3±1.15	4.4±1.38	4.6±0.76	4.9±0.35	4.0±0.6	0.713/0.59
Calcium (mg/dl)	7.3±0.49	7.4±0.26	7.7±0.3	7.3±0.32	7.2±0.28	1.522/0.233

* p<0.05 in comparison to Group 1,

# p<0.05 in comparison to Group 2,

+ p<0.05 in comparison to Group 3,

β p<0.05 in comparison to Group 4. HDL=High-density lipoprotein, VLDL=Very low-density lipoproteins, LDL=Low-density lipoproteins

In CCl_4_-treated rats, CCl_4_ caused a significant elevation of AST, ALT, AP, and total and direct bilirubin, and caused a significant lowering of total protein (p<0.05) (Table-3). TVH, OVOE, and HEM significantly ameliorated these changes. TVH was more potent than OVEO, and HEM was more potent than TVH and OVEO individually (p<0.05). Regarding kidney function and serum electrolytes, CCl_4_ significantly elevated blood urea, creatinine, sodium, potassium, and calcium, while TVH, OVOE, and HEM significantly decreased all these parameters.

**Table-3:** Effect of *Thymus vulgaris* honey, *Origanum vulgar* essential oil and HEM on liver, kidney function, and lipid profile in CCl4-treated rat.

Variables	Treatment groups					F/P value

	Control	CCl4	*Thymus vulgaris* honey	*Origanum vulgar* essential oil	HEM	
AST (U/L)	131.0±3.0	246.0±8.0*	214.0±9.95*#	228.0±7.5*#+	156.0±3.0*#+β	248.785/0.000
ALT (U/L)	124.0±3.0	192.0±2.1*	145.0±7*#	166.0±5.7*#+	121.0±7.6*#+β	191.82/0.00
ALKP (U/L)	137.0±4.5	314.0±3.05*	252.0±5.29*#	288.0±6*#+	166.0±3*#+β	1488.6/0.00
Total protein (g/dl)	6.5±0.07	4.9±1.55*	6±1.03*#	4.9±2.0*#+	6.3±1.5*#+β	1.557/0.22
Total bilirubin (mg/dl)	0.15±0.07	0.96±0.05*	0.36±0.03*#	0.49±0.04*#+	0.28±0.08*#β	149.0/0.00
Direct bilirubin (mg/l)	0.1±0.02	0.51±0.04*	0.3±0.03*#	0.38±0.05*#+	0.21±0.05*#+β	78.0/0.00
Cholesterol (mg/dl)	71.0±10.0	107.0±3.0*	73.0±2.0*#	85.0±3.0*#+	61.0±6.0*#+β	49.177/0.00
Triglycerides (mg/dl)	39.0±6.0	62.0±11.0*	49.0±2.0#	55.0±2.0*	38.0±3.0#β	15.27/0.00
HDL (mg/dl)	22.0±2.0	16.0±1.0*	22.0±2.0#	19.0±1.0*#+	22.0±1.0#β	16.36/0.00
LDL (mg/dl)	41.0±9.0	78.0±4.0*	40.0±9#	58.0±8.0*#+	32.0±5.0#+β	31.66/0.00
VLDL (mg/dl)	8.0±1.0	12.0±2.0	10.0±2.0	11.0±5.0	8.0±1.0	2.285/0.09
Creatinine (mg/dl)	0.34±0.04	0.61±0.05*	0.47±0.06*#	0.50±0.05*#	0.46±0.02*#	20.4/0.00
Urea (mg/dl)	24.0±2.0	42.0±1.0*	33.0±2.0*#	34.0±1.0*#	30.0±1.0*#+β	97.27/0.00
Sodium (mmol/l)	127.0±2.6	162.0±1.15*	140.0±0.71*#	146.0±3.54*#+	134.0±2.6#+β	123.0/0.00
Potassium (mmol/l)	4.3±1.15	8.5±1.54*	6.5±0.7	6.9±1.7*	4.6±0.65#β	9.93/0.0001
Calcium (mg/dl)	7.3±0.49	10.2±0.35*	8.9±0.06*#	8.6±0.26*#	7.9±0.36#+	34.83/0.00

* p<0.05 in comparison to Group 1,

# p<0.05 in comparison to Group 2,

+ p<0.05 in comparison to Group 3,

β p<0.05 in comparison to Group 4. AST=Aspartate aminotransferase, ALT=Alanine aminotransferase, HDL=High-density lipoprotein, VLDL=Very low-density lipoproteins, LDL=Low-density lipoproteins

TVH was more potent than OVOE, and HEM was more potent than TVH and OVOE (p<0.05). Lipid assays revealed that CCl_4_ significantly increased all the lipid parameters and decreased HDL significantly. However, TVH, OVOE, and HEM significantly lowered the elevated lipid parameters as well as elevated the lowered HDL. TVH was more potent than OVOE, and HEM was more potent than TVH and OVOE (p<0.05).

## Discussion

The data presented here demonstrated for the first time the presence of epicatechin gallate in Moroccan TVH and the protective effect of TVH, OVEO, and their combination against CCl_4_ induced liver and kidney damage. TVH was more potent than OVOE to protect against CCl_4_ toxicity, and HEM was more potent than TVH and OVOE individually.

The pH of TVH was 3.90±0.01, which was less than the pH of *Ceratonia siliqua* honey samples (range from 4.17 to 5.05) collected from Morocco [28]. Furthermore, it was less than the pH of TVH collected from other different areas in Morocco; Rachidia 4.51±0.02, Saouira 4.04±0.03, and Zaraphyt 4.36±0.02 [29]. It means that TVH collected from Fez, boulemane, was more acidic. However, the pH value was within the range of pH of French honey (3.46-6.48), Uruguayan honey (3.0-4.3), Portuguese honey (3.98-5.05), and Iranian honey (3.62-6.61) [30-32]. The pH of honey could be affected by the method of extraction and time of storage.

Other compositions, including water content, ash, and electrical conductivity, are almost within the range found in *C. siliqua* honey collected from Morocco [28].

The ash content and electrical conductivity correlated with the mineral content of honey [33]. The ash content was 0.24±0.01%, which is following the standards of ash content in honey used in the EU (ash percentage of honey <0.6%). Furthermore, the ash content of TVH lies in the range of Iranian honey samples (0.041-0.562%) and Indian honey samples (0.03-0.43%) [30,34].

The electrical conductivity of TVH was 449±1.73 mS/cm. According to the EU, the maximum limit value of electrical conductivity for thyme honey is 800 μS/cm [35]. The electrical conductivity of TVH lies in the range of Iranian honey (210 and 1120 μS/cm), Spanish honey (89 and 1213 μS/cm), and Indian honey (330 and 940 μS/cm) [30,36]. It was found that electrical conductivity has a correlation with the mineral content of honey, and it is usually used for routine honey quality control and to determine its botanical origin [33,37].

The honey moisture content is an important criterion to determine the quality of honey. The water content depends on nectar, storage and environmental conditions, and harvest season. TVH moisture was 16.4±0.01%, and it was within the range of Iranian honey moisture (13.97-17%), Portuguese honey moisture (13.1 and 16.9), and within limits (≤20%) set by the EU [30,32,34,35]. The differences observed between the samples might be related to environmental conditions, harvest period, and degree of honey maturity [35].

The mineral content is an essential part of the nutritional values of honey. The results showed that TVH contains necessary minerals that involve in several vital functions of the animals and the human body. It was found that potassium was the most abundant element in the thyme honey. The range of potassium concentration in TVH is almost within the range obtained in other honey types collected from different countries [29,36]. Another study showed that potassium made up 73% of 18 different minerals in ten mono-floral kinds of honey [33]. Furthermore, a positive relationship between mean conductivity and total mineral content and pH and total mineral content was observed [33]. The mineral content is used as a parameter in determining the botanical and geographical origins of honey [16,17].

The Pfund scale value of TVH was 0.33± 0.01, which indicated that the color was dark amber. The color is similar to carob honey and Portuguese honey [29,37]. The color depends on antioxidants, minerals, and pollen content [29].

Phenolic compounds are plant-derived secondary metabolites and transferred through the nectar to the honey. The phenolic compounds can be classified into phenolic acids and flavonoids. The content of phenolic compounds of honey depends on the floral and geographical origins [14].

The value of phenol content in TVH was 74.05±1.21 mg GAE/100 g. It was found that phenol content of 17 commercial honey samples from Morocco varied from 16.38 mg GAE/100g in citrus honey to 92.37 mg GAE/100 in thyme honey from Rachidia [27]. Therefore, the phenol content in TVH was less than that in thyme honey collected from Rachidia, Morocco. Iranian honey showed higher total phenol content (ranged between 193.8 mg GAE/100 g and 3020 mg GAE/100 g) than TVH [30]. The total phenolic content of honey samples collected from Greek ranged from 55 to 92 mg GAE/100 g, and Manuka honey had a phenolic content (71 mg GAE/100g) [38]. Total phenolic content of 12 different Mediterranean floral sources in Jordan ranged between 33.7 mg GAE/100 g and 86.3 mg GAE/100 g [39]. Therefore, the phenolic content of TVH lies within the range of Jordanian honey and Greek honey samples, and it is almost similar to Manuka honey.

With the use of HPLC, the standard compounds were detected at 280 nm because most of the phenolic compounds showed reasonably high absorbance at this value. Five phenolic compounds were identified in TVH extract: Ferulic acid, gallic acid, caffeic acid, epicatechin gallate, and pyrogallol. The major constituent in the sample was epicatechin gallate, with a concentration of 6.91±0.05 mg/100 g. Ferulic acid, gallic acid, and caffeic acid have been reported in Malaysian honey [24]. Twenty-eight samples of thyme honey collected from Greek were analyzed, and a total of 62 compounds were isolated. Thyme honeys contains various compounds such as phenylacetaldehyde, 1-phenyl-2,3-butanedione, 3-hydroxy-4-phenyl-2-butanone, 3-hydroxy-1-phenyl-2-butanone, phenylacetonitrile, and carvacrol [40]. However, no epicatechin gallate was detected. In another study, six phenolic acids (gallic, syringic, benzoic, trans-cinnamic, p-coumaric, and caffeic acids) and five flavonoids (catechin, kaempferol, naringenin, luteolin, and apigenin) were identified in Malaysian Tualang, Gelam, and Borneo tropical honey [41].

Epicatechin gallate was detected for the first time in TVH. Epicatechin gallate was not detected in honey before. It is useful in treating wounds, and it exhibits a potent anti-inflammatory and analgesic effect by lowering levels of plasma prostaglandin E2, tumor necrosis factor-α, interleukin-1β (IL-1β), and IL-6 [42-44]. Therefore, epicatechin gallate in the TVH makes it a potential agent to be used in wounds and inflammation. Furthermore, as epicatechin gallate was not found in other honey, it might be a useful marker for TVH from Morocco.

The value of flavonoid content was 59.34±1.08 mg QE/100 g, and flavonol was 15.08±0.04 mg QE/100 g. Flavonoid content was 0.42 mg QE/100g in citrus honey and 13.962 mg QE/100g in black cumin honey [29]. Therefore, the flavonoid content of TVH was higher than that found in citrus honey as well as black cumin honey.

The total antioxidant capacity of TVH was 163.51±8.55 mg Eq AA/g. The IC_50_ values of thyme honey from Rachidia, Morocco, were 52.85±1.2 mg/mL, and from Saouira, Morocco, were 51.76±1.26 mg/mL [29]. This means that the DPPH scavenging activity of THV was higher than that of thyme honey collected in different areas of Morocco. The IC_50_ values of 21 different types of honey, which were derived from the Olympus mountain area, Greek, ranged from 7.5 to 109.0 mg/mL, while Manuka honey exhibited a weak antioxidant activity (IC_50_; 68.0 mg/mL) in DPPH assay [38]. Therefore, TVH was more potent than Manuka honey as an antioxidant and more than most Greek honey. IC_50_ of Malaysian Tualang, Gelam, and Borneo tropical honey ranged between 5.24±0.40 and 17.51±0.51 mg/mL, and therefore, the antioxidant activity of TVH lies in the same range [41]. Jordanian honey collected in summer from Umm Alyanabea location has IC_50_=24.5 mg/mL, which is weaker than TVH as an antioxidant. The use of DPPH to evaluate the antioxidant activity is based on determining the ability of TVH to act as a hydrogen or electron donor in the conversion of DPPH in its reduced form (DPPH-H).

The GC/MS analysis of OVOE revealed two major compounds: Carvacrol (48.38%) and thymol (26.55%), which agrees with another study [45]. Furthermore, the compounds demonstrated in the OVOE were similar to *O. vulgare* from Morocco and other countries, which showed that the major compound was carvacrol [25,46]. The percentage of carvacrol and thymol in the OVOE sample is twice as high as those identified in OVOE from other Morocco areas [25]. The difference might be related to the method of isolation, geographic origin, and season of harvesting [47]. It has been reported that 37 compounds were identified in the leaf-flower oils and included carvacrol (30.73%), thymol (18.81%), P-cymene (10.88%), caryophyllene (7.73%), and 3-carene (4.06%) [48]. Therefore, carvacrol and thymol contents were less than those found in the present study. In another study, seven components were identified in OVEO; a-thujene, myrcene, a-terpinene, o-cymene, γ-terpinene, thymol, and carvacrol; thymol content was 45%, and carvacrol content was 37.4% [49].

The IC_50_ value of OVEO prepared from the aerial part (stem and leaves) of the plant was 0.30±0.02 mg/mL. It is almost similar to the results of the IC50 values of the Chinese OVEO prepared from leaves/flower (0.332±0.040 mg/mL) and the plant root ( 0.357±0.031 mg/mL), but less than IC_50_ values of essential oil prepared from the plant stem (0.501±0.029 mg/mL) [48]. It was found that IC_50_ of OVEO ranged from 0.32 mg/mL to 0.76 mg/mL, depending on incubation time [21]. However, the IC50 value of OVEO was less than that of TVH (10.85±0.02 mg/mL), which means that the DPPH scavenging activity of OVEO was higher than that of TVH.

The results showed that CCl_4_ significantly increases serum levels of AST, ALT, and ALP compared to the control group. However, administration of TVH, OVEO, or their combination to the animals with CCl_4_-induced hepatotoxicity decreased significantly liver enzymes compared to the CCl_4_ treated group. This finding showed the hepatoprotective effect of TVH, OVOE, and HEM. Interestingly, HEM showed a more profound hepatoprotective effect than TVH or OVEO. The potentiation of HEM effect might be due to the synergistic activity of natural ingredients in TVH and OVEO. It was found that *O. vulgare* leaf extract has significant protection against CCl_4_-induced hepatotoxicity in a dose-dependent manner. It lowered serum ALT, ALP, and AST levels and elevated Glutathione S-transferase, catalase, superoxide dismutase, glutathione peroxidase, glutathione reductase, and GSH levels in liver tissue [50]. Furthermore, others and we found that honey could protect the liver and kidney after CCl_4_ intoxication by normalizing liver enzymes and kidney function [8-10].

CCl_4_ significantly increased TG levels, cholesterol, and LDL and decreased serum levels of HDL compared to the control group. TVH, OVEO, and HEM markedly ameliorated these effects. This finding agrees with the impact of Sidr honey on lipid profile in CCl_4_ toxicity [8]. In CCl_4_-untreated rats, TVH or OVEO has no significant effect on lipid profile; however, their combination caused a considerably lower TC, LDL, and VLDL and considerable HDL elevation. This result is most likely due to an additive or synergistic effect of active ingredients in TVH and OVEO. This finding makes their combination a good candidate for prophylaxis of dyslipidemia and cardiovascular diseases.

CCl_4_ significantly increased the blood level of creatinine and urea. The mechanism of CCl_4_ induced renal toxicity is almost the same as that of the liver. Furthermore, CCl_4_ has a strong affinity to the kidney cortex because it contains a high level of cytochrome P-450 [51]. The administration of TVH, OVEO, and their mixture significantly decreased the elevated levels of creatinine, urea, sodium, potassium, and calcium; the effect was more pronounced with HEM. Recently, we have found that CCl_4_ causes a significant elevation of oxidative stress markers in the blood and kidney tissues, which were markedly alleviated by Carob honey’s administration (Unpublished).

TVH has a high content of epicatechin galatte and ferulic acid, while OVOE contains carvacrol and thymol. Epicatechin gallate has anti-inflammatory, analgesic, and wound healing properties [42,43]. Ferulic acid effectively reduced lipid disorders in the plasma, liver, and kidney of CCl_4_-treated rats [52]. Carvacrol is a phenolic monoterpenoid found in essential oils of *O. vulgare*, *T. vulgaris*, and other plants. A recent review showed that carvacrol has antioxidants, antimicrobial, and anti-inflammatory activity [53]. The mechanism of action of TVH, OVEO is most likely due to antioxidant capacity and anti-inflammatory activity, which are dependent on their active chemical ingredients, particularly thymol, carvacrol, epicatechin galatte, and ferulic acid.

## Conclusion

**Figure-2:**
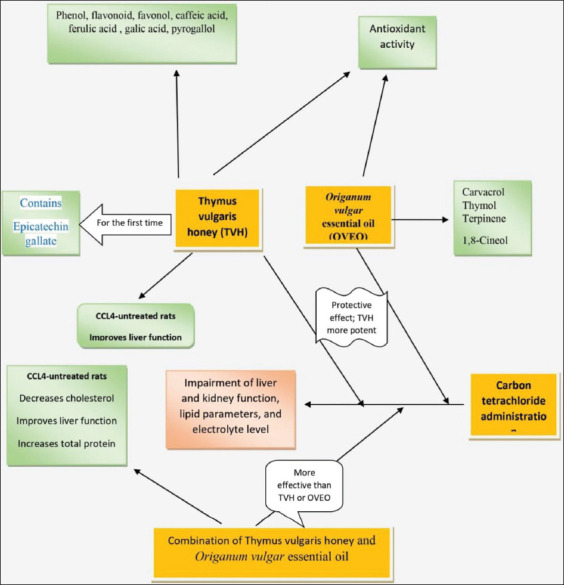
Main effects of thymus honey and Origanum vulgar essential oil.

The main finding of this study is summarized in Figure-2. Interestingly, the data showed for the first time that TVH contains a high level of epicatechine galatte, which makes it a marker for TVH. The analysis of the OVEO confirms the presence of two major compounds: carvacrol and thymol. HVT and OVEO exhibited a strong antioxidant activity and have a high capacity to scavenge the free-radical DPPH. Furthermore, TVH, OVEO, and HEM showed a significant protective effect against CCl_4_-induced liver and kidney damage, electrolyte disturbances, and lipid disorder. Although the ability of OVEO to scavenger DPPH was higher than TVH; TVH was more potent than OVEO to protect against CCl_4_ toxicity. Therefore, TVH, OVEO, and HEM are significant interventions to protect against chemical toxicity and might be useful in other causes of kidney and liver failure and dyslipidemia. While TVH and OVEO did not significantly affect kidney and liver function in CCl_4_ non-treated rats, their combination causes a substantial improvement in lipid profile and kidney and liver function. Therefore, the use of HEM in normal individuals might improve the lipid profile and liver and kidney function and protect against cardiovascular disease. These findings are exciting and will stimulate further preclinical and clinical studies in this growing field.

## Authors’ Contributions

HI, AAb, AAbd, and BL designed the experimental protocols and participated in the practical work and writing of the paper. NA analyzed the data and results and wrote the manuscript for publication. TA did the statistical analysis. All authors read and approved the final manuscript.
